# Specificity analysis of sera from breast cancer patients vaccinated with MUC1-KLH plus QS-21

**DOI:** 10.1038/sj.bjc.6990288

**Published:** 1999-04

**Authors:** S Adluri, T Gilewski, S Zhang, V Ramnath, G Ragupathi, P Livingston

**Affiliations:** Department of Medicine, Memorial Sloan-Kettering Cancer Center, 1275 York Avenue, New York, NY, 10021, USA

**Keywords:** MUC1, keyhole limpet haemocyanin (KLH), QS-21, vaccine, breast cancer, antibodies

## Abstract

The mucin MUC1 is expressed on breast cancers in an underglycosylated form compared to normal tissues and is therefore a potential target for cancer immunotherapy. MUC1 contains multiple tandem repeats of the 20 amino acid (aa) peptide (VTSAPDTRPAPGSTAPPAHG). The APDTRPA epitope is particularly immunogenic since it is recognized by a variety of murine monoclonal antibodies and by some sera and cytotoxic T-cells from unimmunized patients with epithelial cancers. We have prepared a 30 aa peptide (C)VTSAPDTRPAPGSTAPPAHGVTSAPDTRPA with cysteine at the N-terminal end, and used the cysteine for chemical conjugation to keyhole limpet haemocyanin (KLH). Six breast cancer patients immunized with this conjugate plus the immunological adjuvant QS-21 have all produced high titre (by ELISA) IgG and IgM antibodies against the 30 aa MUC1 peptide, but these sera reacted moderately, or not at all, with MUC1-positive tumour cells. To understand this specificity better, we prepared a series of smaller peptides to determine the epitopes recognized by these immune sera in inhibition assays. Only peptides containing APDTRPA at the C-terminal end were able to completely inhibit ELISA reactivity for the full 30 aa peptide. No sera were completely inhibited by APDTR, APDTRP, PDTRPA or any other peptides that did not contain the full APDTRPA epitope. Remarkably, sera from all six patients recognized this same epitope and were completely inhibited by only this epitope. The specificity of these sera (1) primarily for C-terminal APDTRPA, and the absence of this epitope at the C-terminal end of any tumour mucins, and (2) the N-terminal APDTRPA alanine, which is normally buried in the β turn MUC1 assumes in its secondary structure may explain the moderate to weak reactivity of these high titer sera against MUC1-positive tumour cells. © 1999 Cancer Research Campaign


British Journal of Cancer (1999) **79**(11/12), 1806–1812
© 1999 Cancer Research Campaign
Article no. bjoc.1998.0288

### Specificity analysis of sera from breast cancer patients vaccinated with MUC1-KLH plus QS-21

**S Adluri, T Gilewski, S Zhang, V Ramnath, G Ragupathi and P Livingston**

Department of Medicine, Memorial Sloan-Kettering Cancer Center, 1275 York Avenue, PO Box 113, New York, NY 10021, USA

**Summary** The mucin MUC1 is expressed on breast cancers in an underglycosylated form compared to normal tissues and is therefore a potential target for cancer immunotherapy. MUC1 contains multiple tandem repeats of the 20 amino acid (aa) peptide (VTSAPDTRPAPGSTAPPAHG). The APDTRPA epitope is particularly immunogenic since it is recognized by a variety of murine monoclonal antibodies and by some sera and cytotoxic T-cells from unimmunized patients with epithelial cancers. We have prepared a 30 aa peptide (C)VTSAPDTRPAPGSTAPPAHGVTSAPDTRPA with cysteine at the N-terminal end, and used the cysteine for chemical conjugation to keyhole limpet haemocyanin (KLH). Six breast cancer patients immunized with this conjugate plus the immunological adjuvant QS-21 have all produced high titre (by ELISA) IgG and IgM antibodies against the 30 aa MUC1 peptide, but these sera reacted moderately, or not at all, with MUC1-positive tumour cells. To understand this specificity better, we prepared a series of smaller peptides to determine the epitopes recognized by these immune sera in inhibition assays. Only peptides containing APDTRPA at the C-terminal end were able to completely inhibit ELISA reactivity for the full 30 aa peptide. No sera were completely inhibited by APDTR, APDTRP, PDTRPA or any other peptides that did not contain the full APDTRPA epitope. Remarkably, sera from all six patients recognized this same epitope and were completely inhibited by only this epitope. The specificity of these sera (1) primarily for C-terminal APDTRPA, and the absence of this epitope at the C-terminal end of any tumour mucins, and (2) the N-terminal APDTRPA alanine, which is normally buried in the β turn MUC1 assumes in its secondary structure may explain the moderate to weak reactivity of these high titer sera against MUC1-positive tumour cells.

**Keywords:** MUC1; keyhole limpet haemocyanin (KLH); QS-21; vaccine; breast cancer; antibodies

Mucins are highly glycosylated proteins that are expressed in cancers of epithelial origin in an underglycosylated form. MUC1 is such a mucin and is the most abundant mucin in breast cancers (Ceriani et al, 1983; Sekine et al, 1985; Gum et al, 1989, 1990; Lan et al, 1990; McKenzie and Xing 1990; Strauss et al, 1992). In normal tissues, but not cancers, MUC1 is highly glycosylated and its expression is largely restricted to the apical surface of luminal cells, two factors which combine to make MUC1 in normal tissues inaccessible to the immune system. Thus the underglycosylated MUC1 protein on cancer cells is a potential target for vaccine therapy (Girling et al, 1989; Hilkens et al, 1989; Sell, 1990; Devine et al, 1991; Itzkowitz et al, 1991; Jerome et al, 1991).

MUC1 mucin is a high molecular weight molecule with multiple tandem repeats of peptide (VTSAPDTRPAPGSTAPPAHG). The most immunogenic position is APDTRPA which includes the epitope recognized by a variety of monoclonal antibodies (mAbs) (Xing et al, 1992), normal sera (von Mensdorff-Pouilly et al, 1996), and cytotoxic T-cells (Nieves et al, 1995). Zhang et al (1996) were able to vaccinate mice with MUC1 peptide containing 1.5 tandem repeats conjugated to keyhole limpet haemocyanin (KLH) and mixed with QS-21, and were able to induce high titre antibody (but no evidence of T-cell immunity) against MUC1 and tumour cells expressing MUC1. Moreover, these vaccinations were able to confer protection in these mice when they were challenged with MUC1-expressing tumour cells.

*Received 16 March 1998*
*Revised 28 July 1998*
*Accepted 10 August 1998*

*Correspondence to:* P Livingston

Consequently, patients with AJCC stage I–III breast cancer and rising CA15.3 or CEA markers, and patients with AJCC stage IV breast cancer who were currently free of disease, were vaccinated with a MUC1-KLH plus QS-21 vaccine (Gilewski et al, 1998). The vaccine induced high titres of IgM and IgG antibodies against the MUC1 peptide. However, the IgM antibodies from only four of the six patients reacted moderately with tumour cells expressing the MUC1 antigen and IgG antibodies from only three of six patients reacted (weakly) with tumour cells. The goal of the study described here was to determine the precise specificity of these MUC1 immune sera in hopes that it would lead us to an explanation for why these high titer MUC1 antisera showed relatively weak reactivity against MUC1 on tumour cells.

### MATERIAL AND METHODS

### Materials

MUC1 was synthesized using an Applied Biosystems Model 431A automated peptide synthesizer by the MSKCC microchemistry core facility. The amino acid (aa) sequence is (c)VTSAPDTRPAPGSTAPPAHGVTSAPDTRPA. This represents 1.5 repeats of the 20 aa MUC1 peptide, with the repeated section representing the most immunogenic epitope. Cysteine was added to the N-terminal carboxyl end to facilitate attachment to a carrier protein. Peptides used for inhibition assays were made by Research Genetics (Huntsville, AL, USA). KLH used as a carrier protein was purchased from PerImmune Inc. (Rockville, MD, USA). QS-21 adjuvant, a purified saponin fraction, was obtained from Aquila Biopharmaceuticals Inc. (Farmingham, MA, USA). Monoclonal antibody HMFG-2 against MUC1 was kindly provided by Dr

**Table 1** Serological results of six breast cancer patients vaccinated with MUC1 (30 aa)-KLH conjugate plus QS-21

| Patient | Peak ELISA titre against MUC1 (30 aa) | | FACS analysis on MCF-7 cell line | | | | | | | |
|---|---|---|---|---|---|---|---|---|---|---|
| | IgM | IgG | IgM | | | | IgG | | | |
| | | | Pre-vaccination | | Post-vaccination | | Pre-vaccination | | Post-vaccination | |
| | | | Positive cells (%) | Mean fluorescence intensity | Positive cells (%) | Mean fluorescence intensity | Positive cells (%) | Mean fluoresence intensity | Positive cells (%) | Mean fluorescence intensity |
| 1 | 2560 | 2560 | 9.79 | 11.14 | 64.71 | 33.28 | 10.63 | 9.43 | 38.11 | 16.86 |
| 2 | 1280 | 2560 | 10.92 | 31.23 | 25.5 | 49.98 | 10.4 | 4.92 | 24.28 | 7.85 |
| 3 | 20 480 | 2560 | 10.58 | 12.84 | 84.28 | 66.41 | 9.7 | 9.93 | 34.06 | 17.20 |
| 4 | 2560 | 2560 | 9.72 | 28.51 | 2.49 | 17.49 | 10.93 | 7.24 | 18.41 | 10.36 |
| 5 | 2560 | 10 240 | 9.95 | 10.03 | 91.36 | 45.2 | 10.80 | 4.47 | 16.57 | 5.62 |
| 6 | 20 480 | 10 240 | 9.38 | 15.38 | 59.67 | 41.77 | 11.21 | 4.56 | 21.34 | 5.81 |

Joyce Taylor-Papadimitrou (Taylor-Papadimitrou et al, 1981), and the human breast tumour cell line MCF-7 (Soule et al, 1973) was obtained from Dr Neil Rosen (Memorial Sloan-Kettering Cancer Center).

### Conjugation of MUC1 peptide to KLH

Covalent attachment to KLH was achieved with m-malemido-benzoyl-*N*-hydroxysuccinimide ester (Pierce Co., Rockford, IL, USA) which couples the terminal cysteine on the MUC1 peptide to amino groups on KLH. The MBS–dimethylformamide is mixed with KLH and the unconjugated MBS eliminated by passage over a G25 sephadex column. The malemide-activated KLH was then added to the MUC1 peptide and, following a 3-h incubation, the free peptide was eliminated using a 30 000 MW centriprep filter as previously described (Amicon Inc., Beverly, MA, USA) (Zhang et al, 1996). The resultant conjugant was washed and tested for sterility, purity and safety. The ratio of MUC1 peptide molecules per KLH molecule was 540 to 1.

### Patients and treatment schedule

Sera from the initial six patients in a larger MUC1 vaccination trial were studied. Patients with AJCC stage I–III breast cancer and rising CA15.3 or CEA markers, and patients with AJCC stage IV breast cancer who were currently free of disease, were vaccinated. Accrual of patients was done under an IRB-approved protocol. MUC1-KLH conjugate containing 100 μg of MUC1 plus 100 μg QS-21 was administered subcutaneously at weekly intervals for three doses, followed by a 4-week break and then a fourth vaccination. This was followed by a 3-month break and a fifth vaccination.

### Enzyme-linked immunosorbent assay (ELISA)

ELISAs were performed using alkaline–phosphatase conjugated goat anti-(human) IgM (Kierkegaard and Perry Labs, Gaithersburg, MD, USA) as previously described (Zhang et al, 1996). To detect IgG against MUC1, an unlabelled mouse anti-human IgG (Southern Biotech, Birmingham, AL, USA) was used followed by alkaline–phosphatase labelled goat anti-mouse IgG (Southern Biotech). A total of 0.2 μg of MUC1 peptide was plated using carbonate buffer (pH 10) into Nunc (Nunc, Denmark) 96-well plates. Absorbance was measured at 414 nm and the highest serum dilution with an absorbance of at least 0.100 was defined as the antibody titre. Plates were incubated before measuring absorbance, at 37°C for approximately 20 min. Inhibition ELISAs were performed to determine more sensitively the specificity of the antibodies. For these assays, the designated amount of peptide was incubated with patient sera for 1 h and the mixture then tested on the 30 aa MUC1 peptide by ELISA. Percentage inhibition was calculated on the basis of the difference in absorbance from the uninhibited serum.

% inhibition = *absorbance of uninhibited serum – absorbance of inhibited serum*/ absorbance of uninhibited serum

In Figures 1 and 2, error bars have been calculated and added to account for variability between experiments and reproducibility of the data. For each peptide at each of the concentrations, the mean value of percent inhibition within four different repeats of experiments was calculated and then error bars were calculated to reflect the variability of the mean at that concentration, as previously described (Woolson, 1987).

### Flow cytometry

Tumour cells ($1 \times 10^7$) were incubated with 20 μl of 1:10 diluted sera or 1:2 diluted mAb supernatant on ice for 30 min. After washing with 3% fetal calf serum (FCS), the cells were incubated with 20 μl of 1:25 diluted fluorescence in situ hybridization (FITC)-labelled goat anti-human IgM or IgG (Southern Biotech. Assoc. Inc., Birmingham, AL, USA). The positive population of cells was quantitated by flow cytometry (Becton Dickinson FACSCAN, Sanjose, CA, USA). Pre-vaccination sera was used to set the gate at 10% background for each post-vaccination sample.

### RESULTS

### Direct tests

Antibody titres against MUC1 were measured by ELISA. High titre IgM and IgG antibodies against MUC1 peptide were induced in all patients. Pre-vaccination serum titres were low in all patients, except patient 6 who had a pretreatment IgM titre of

*© Cancer Research Campaign 1999* 

**Table 2** Inhibition of anti-MUC1 IgM reactivity in patient sera with MUC1 (30 aa) and other synthetic peptides

| | Patients | | | | | | |
|---|---|---|---|---|---|---|---|
| **Peptide** | **1** | **2** | **3** | **4** | **5** | **6** | **Positive control HMFG-2** |
| 30 aa (1.5 repeats) | 4+ | 3+ | 4+ | 4+ | 3+ | 4+ | 4+ |
| PAHGVTSAPDTRPA | 4+ | 4+ | 3+ | 4+ | 4+ | 3+ | 3+ |
| APDTRPA | 4+ | 3+ | 3+ | 4+ | 4+ | 3+ | 3+ |
| VTSAPDTRPAPGS | 1+ | 0 | 0 | 0 | 0 | 0 | 4+ |
| PDTRPAPGSTAPPAH | 0 | 0 | 0 | 0 | 0 | 0 | 4+ |
| PAPGSTAPPAHGVTS | 0 | 0 | 0 | 0 | 0 | 0 | 0 |
| PAHGVTS | 0 | 0 | 0 | 0 | 0 | 0 | 0 |
| TSAPDTR | 0 | 0 | 0 | 0 | 0 | 0 | 0 |
| APDTR | 0 | 0 | 0 | 0 | 0 | 0 | 0 |

Grading scale: percent inhibition of 85–90% was given a grade of 4+, 70–85% was a 3+, 40–70% was 2+, 20–40% was 1+, below 20% was 0.

**Table 3** Inhibition of anti-MUC1 IgG reactivity in patient sera with MUC1 (30 aa) and other synthetic peptides

| | Patients | | | | | | |
|---|---|---|---|---|---|---|---|
| **Peptide** | **1** | **2** | **3** | **4** | **5** | **6** | **Positive control HMFG-2** |
| 30 aa (1.5 repeats) | 4+ | 4+ | 4+ | 4+ | 4+ | 4+ | 4+ |
| PAHGVTSAPDTRPA | 4+ | 4+ | 3+ | 4+ | 4+ | 4+ | 2+ |
| APDTRPA | 3+ | 3+ | 3+ | 4+ | 4+ | 4+ | 4+ |
| VTSAPDTRPAPGS | 2+ | 1+ | 1+ | 2+ | 2+ | 2+ | 4+ |
| PDTRPAPGSTAPPAH | 0 | 0 | 0 | 1+ | 1+ | 0 | 4+ |
| PAPGSTAPPAHGVTS | 0 | 0 | 0 | 1+ | 1+ | 1+ | 0 |
| PAHGVTS | 0 | 0 | 0 | 0 | 0 | 0 | 0 |
| TSAPDTR | 0 | 0 | 0 | 0 | 1+ | 1+ | 0 |
| APDTR | 0 | 0 | 0 | 0 | 1+ | 1+ | 0 |

Grading scale: percent inhibition of 85–90% was given a 4+ grade, 70–85% was 3+, 40–70% was 2+, 20–40% was 1+, below 20% was 0.

1:160 (see Table 1). MCF-7 cells, which express MUC1 antigen, were also used as targets in FACS analysis (Table 1) to determine whether the induced anti-MUC1 antibodies were reactive with the cell surface. Patients 1, 3, 5 and 6 showed moderately increased IgM reactivity after immunization. Weak anti-MUC1 IgG reactivity against MCF-7 cells was induced in patients 1, 2 and 3. HMFG-2, the positive control MUC1 mAb, reacted strongly with 75% of MCF-7 cells.

### Inhibition tests

Sera from immunized patients were incubated (inhibited) with various amounts of small MUC1 peptides. The ELISA results obtained with sera (inhibited or not) from patient 2 are shown in Figures 1 and 2. Complete inhibition of all IgM and IgG reactivity was achieved with the APDTRPA peptide, or longer peptides containing the full APDTRPA epitope at the C-terminal position. Partial inhibition of IgM reactivity was seen with the peptide containing APDTRPA in mid peptide. No inhibition was seen when the full APDTRPA was not present. Tables 2 and 3 summarize the IgM and IgG inhibition results for all six patients. Table 2 shows that the specificity of induced anti-MUC1 IgM antibodies was almost exclusively for a terminal APDTRPA epitope in all six patients. Only serum of patient 1 showed slight inhibition of IgM reactivity with peptide when the APDTRPA epitope was in mid peptide.

Table 3 shows the specificity of the induced anti-MUC1 IgG antibodies. Here again strong inhibition was seen with the 30 aa MUC1 peptide and other peptides with a terminal APDTRPA epitope, in all six patients. However, some inhibition was also seen when the APDTRPA was present in the middle of the peptide. In addition, sera of patients 5 and 6 showed 1+ inhibition with a peptide consisting of only the APDTR epitope, and sera of patients 4, 5 and 6 showed 1+ inhibition with a peptide that had no APDTRPA sequence. Monoclonal antibody HMFG-2 was inhibited with peptides that have APDTRPA epitope regardless of its location. In addition, complete inhibition of HMFG-2 reactivity was also possible with PDTRPAPGSTAPPAH, unlike any of the immune sera, which all required the full APDTRPA epitope for complete inhibition.

### DISCUSSION

The structure of the MUC1 core protein consists of an *N*-terminal protein sequence, followed by a variable number of tandem repeats (VNTR) of the 20 aa peptide and, finally, the transmem-

*© Cancer Research Campaign 1999*

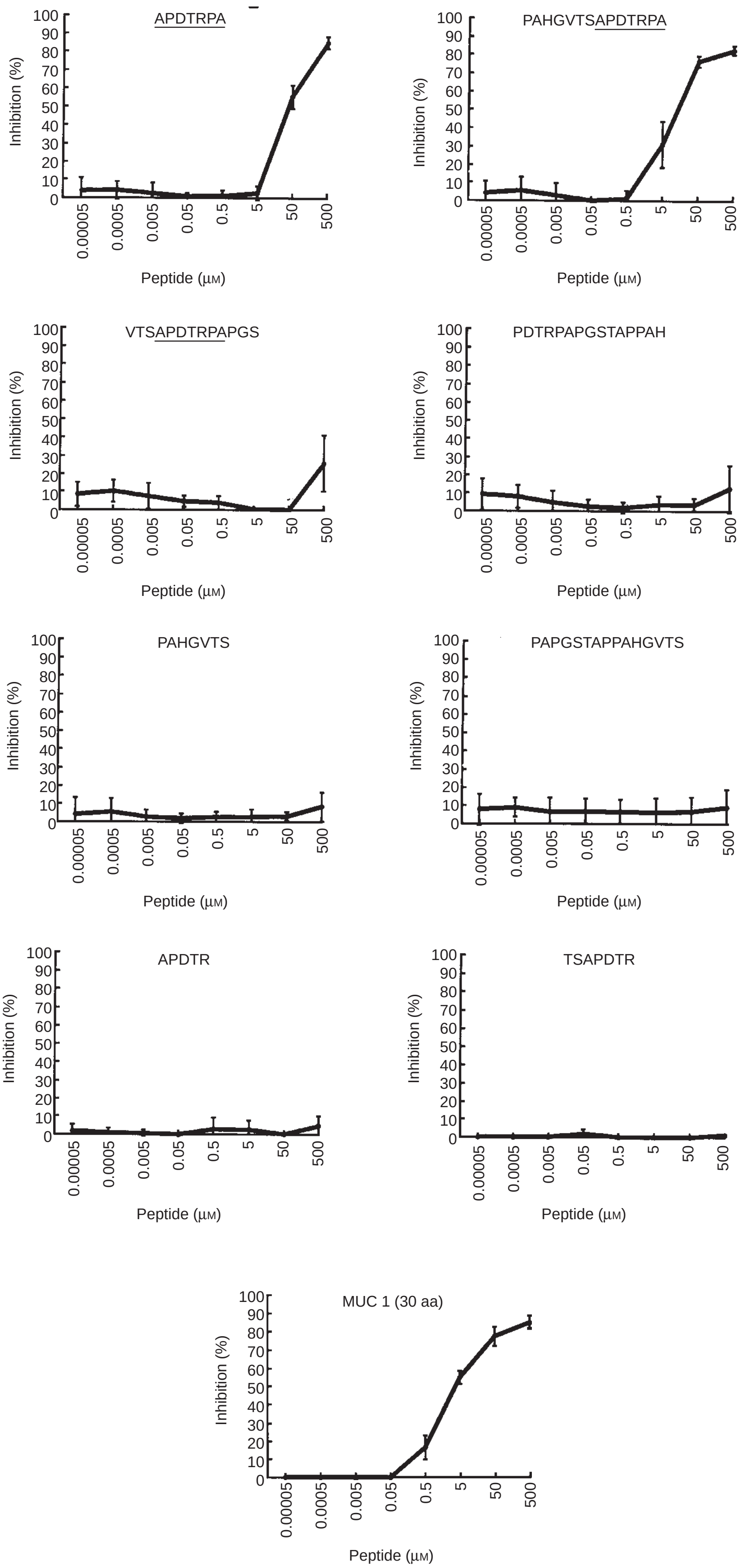

**Figure 1** Inhibition of anti-MUC1 IgM reactivity in post-immunization serum of patient 2. The inhibitory peptides were VTSAPDTRPAPGS, PDTRPAPGSTAPPAH, PAPGSTAPPAHGVTS, PAHGVTSAPDTRPA, PAHGVTS, APDTRPA, APDTR, TSAPDTR and MUC1 (30 aa). Peptides were used at various concentrations to inhibit anti-MUC1 IgM reactivity and then tested by ELISA to measure percent inhibition. MUC1 peptide was coated in each well at 0.2 μg per well. The serum was used at a dilution of 1:80

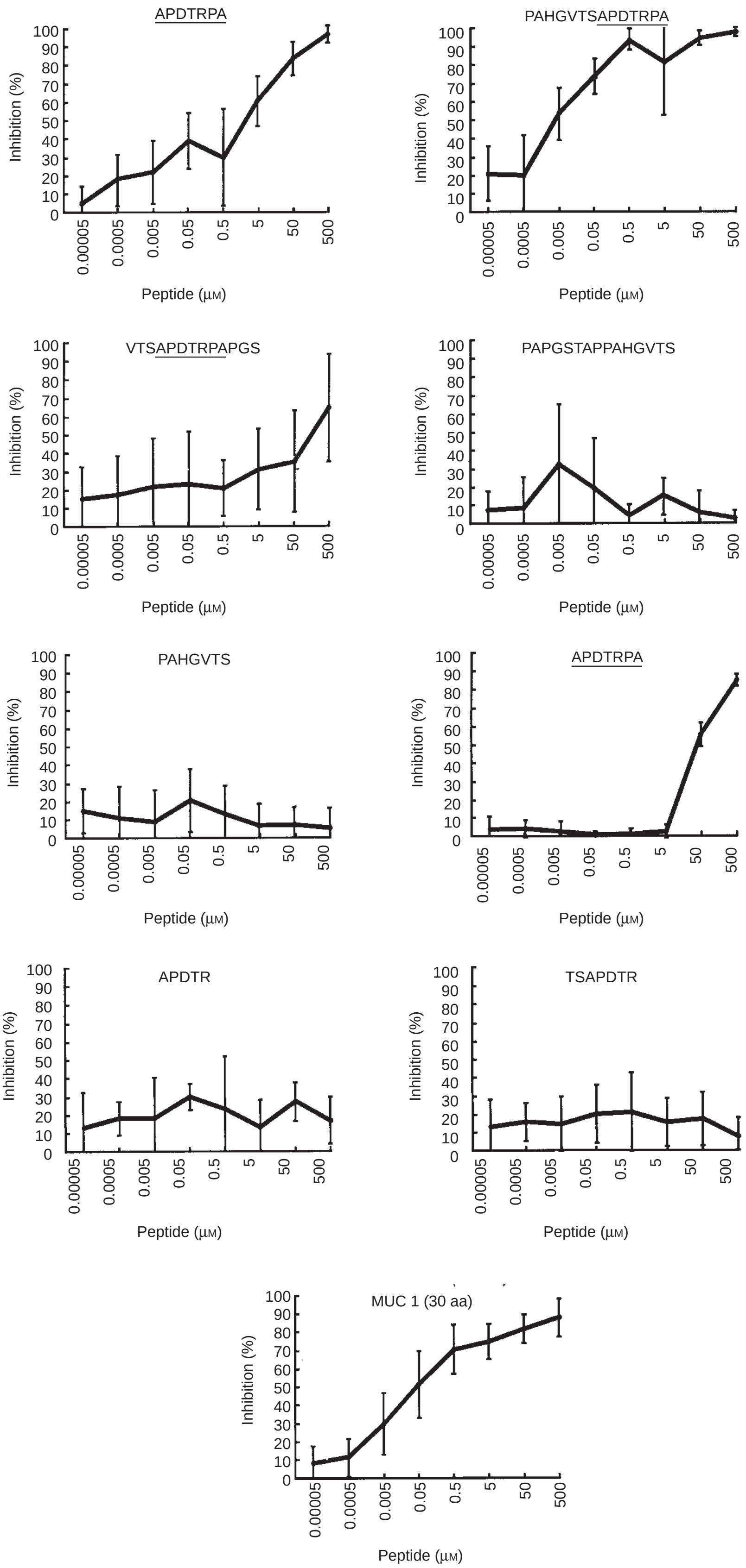

**Figure 2** Inhibition of anti-MUC1 IgG reactivity in post-immunization serum of patient 2. The inhibitory peptides were VTS<u>APDTRPA</u>PGS, PDTRPAPGSTAPPAH, PAPGSTAPPAHGVTS, PAHGVTS<u>APDTRPA</u>, PAHGVTS, <u>APDTRPA</u>, APDTR, TSAPDTR and MUC1 (30 aa). Peptides were used at various concentrations to inhibit anti-MUC1 IgG reactivity and then tested by ELISA to measure percent inhibition. MUC1 peptide was coated in each well at 0.2 μg per well. The serum was used at a dilution of 1:320

 *© Cancer Research Campaign 1999*

brane region and cytoplasmic tail (Price et al, 1990). The VNTR region seems to be the most immunogenic region with the APDTRPA sequence being especially immunogenic. We have synthesized a synthetic peptide 30 amino acids long that contains 1.5 tandem repeats. This MUC1 peptide has two APDTRPA sequences: one toward the $–NH_2$-terminal (but not at the terminal end) and the other at the –COOH-terminal. KLH is conjugated to the $–NH_2$-terminal. Inhibition results with high titre immune sera drawn from breast cancer patients after vaccination indicated that the anti-MUC1 IgM antibodies generated against this vaccine were inhibited specifically and exclusively with peptides that have the full APDTRPA epitope present terminally. The IgG anti-MUC1 antibodies also reacted preferentially with the terminal APDTRPA epitope, but weak reactivity was also seen when the APDTRPA epitope was present in the middle of the peptide and with other portions of the MUC1 peptide.

The antibodies induced in breast cancer patients against this synthetic MUC1 fail to react strongly with mucin on tumour cells. Comparing the reactivity of monoclonal antibody HMFG-2 to these MUC1 anti-sera, HMFG-2 was inhibited by PDTRPA peptide regardless of whether it was expressed terminally or in the middle of the peptide, and HMFG-2 reacted strongly with tumour mucins. Studies of the secondary structure of the protein mucin core of MUC1, suggest that the protein undergoes a β turn which results in the PDTRPAP epitope expressed at the surface of the mucin molecule where it is accessible to antibodies while much of the rest of the VNTR repeat lies buried within the helix (Price, 1988; Price et al, 1990; Price and Tendler, 1993).

The goal of our MUC1 vaccination programme was to induce antibodies that react well with both the synthetic immunogen and tumour cells, like mAb HMFG-2. Based on the results presented here, hypotheses for the relatively modest reactivity of our immune sera with tumour MUC1 are first that the N-terminal alanine, in APDTRPA is not accessible in natural mucins. This alanine is necessary for recognition by our immune sera, but not by HMFG-2. Second, in addition to making the N-terminal alanine of APDTRPA inaccessible, the secondary structure with its β turns assumed by natural MUC1 may have other consequences in terms of the way the individual amino acids in APDTRPA are exposed or the way multiple APDTRPA epitopes in close proximity are recognized by the immune system. Third, the sequence RPA must be at the C-terminal end for strong recognition by our sera, but this is not necessary for HMFG-2 and never occurs in tumour MUC1 (Price et al, 1990). The MUC1 vaccine tested here contained two APDTRPA epitopes, but the APDTRPA at the –COOH-terminal may have been the only one exposed to the immune system, since the other APDTRPA epitope was adjacent to the conjugation site and may have been obstructed due to its proximity to KLH.

We have addressed these three hypotheses with two responses. First, by constructing a 32 aa MUC1 peptide containing two copies of the APDTRPA repeat, neither of which is at the C-terminal end, to force the immune system to recognize the APDTRPA epitope in the middle of the peptide as it occurs naturally. Second, by constructing a 106 aa MUC1 peptide which should naturally assume a β helix configuration similar to the proposed configuration on tumour cells. This should force the immune system to focus on multiple copies of the 20 aa repeat, to see PDTRPA and other potential epitopes in a more natural configuration. In both cases the peptides will be linked to KLH and mixed with QS-21 and the vaccines administered to breast cancer patients in the adjuvant setting. Supporting this approach, Karanikas et al (1997), have vaccinated patients with a 106 aa MUC1 peptide conjugated to mannan (polymanose). They have reported that most of the antibodies in these studies recognized non-APDTRPA epitopes such as STAPPAHG and PAPGSTAP (Karanikas et al, 1997). It therefore seems possible that vaccination with a longer peptide might result in recognition of additional epitopes.

A fourth hypothesis, that the peptide should be partially or completely glycosylated before the proper secondary structure is assumed, will be more difficult to address. There are differing points of view on the proper carbohydrate epitopes and glycosylation sites (Stadie et al, 1995; Karsten et al, 1998), preparation of sufficient glyosylated MUC1 for clinical trials is difficult, and the murine response to vaccination with the same MUC1-KLH vaccine described in this study was high titre IgM and IgG antibodies that reacted strongly with MCF-7, so experiments in the mouse will not be helpful (Zhang et al, 1996). In addition, Apostolopoulos et al (1998) have suggested that presence of anti-*gal* antibodies, which are present in humans, but not the mouse, affects the immune response against MUC1. Consequently, only trials in humans can identify the correct hypothesis and the appropriate vaccine.

### ACKNOWLEDGEMENTS

This work is supported by a grant from NIH (POICA33049).

### REFERENCES

Apostolopoulos V, Osinski C and McKenzie IFC (1998) MUC1 cross-reactive galα (1,3) gal antibodies in humans switch immune responses from cellular to humoral. *Nature Med* **4**: 315–320

Ceriani RL, Peterson JA, Lee JY, Moncada R and Blank EW (1983) Characterization of cell surface antigens of human mammary epithilial cells with monoclonal antibodies, prepared against human milk fat globule. *Somat Cell Genetics* **9**: 415–427

Devine PL, Clark BA, Birell GW, Layton GT, Ward BG, Alegood PF and McKenzie IFC (1991) The breast tumor-associated epitope defined by MoAb 3E1.2 is an O-linked mucin carbohydrate containing N-glycolylneuraminic acid. *Cancer Res* **51**: 5826–5836

Gilewski T, Dickler M, Adluri R, Zhang S, Houghton A, Norton L and Livingston PO (1998) Vaccination of high risk breast cancer patients (PTS) with MUC-1 (32aa) keyhole limpet hemocyarin (KLH) conjugate plus QS-21: Preliminary results. *American Society of Clinical Oncology* **17**: 16–70

Girling A, Bartkova J, Burchell J, Gendler S, Gillet C and Taylor-Papadimitrou JA (1989) Core protein epitope of the polymorphic epithelial mucin detected by MoAb SM-3 is selectively expressed in a range of primary carcinomas. *Int J Cancer* **43**: 1072–1076

Gum JR, Byrd JC, Hicks JW, Tonbara NW, Lamport DTA and Kim TS (1989) Molecular cloning of human intestinal mucin cDNAs. Sequence analysis and evidence for genetic polymorphism. *J Biol Chem* **264**: 6480–6487

Gum JR, Hicks JW, Swallow DM, Lagace RL, Byrd JC, Lamport DTA, Siddiki B and Kim YS (1990) Molecular cloning of cDNAs derived from a novel human intestinal mucin gene. *Biochem Biophys Res Commun* **171**: 407–415

Hilkens J, Buys F and Ligtenberg M (1989) Complexity of MAM-6, an epithelial Sialomucin associated with carcinomas. *Cancer Res* **49**: 786–793

Itzkowitz SH, Kjeldsen T, Friera A, Hakamoi S, Yang US and Kim YS (1991) Expression of Tn, Sialosyl Tn, and T antigens in human pancreas. *Gastroenterology* **100**: 1691–1700

Jerome KR, Barnd DL, Bendt KM, Boyer CM, Taylor-Papadimitrou J, McKenzie IFC, Bast RC Jr and Finn OJ (1991) Cytotoxic T-lymphocytes derived from patients with breast adenocarcinoma recognize an epitope present on the protein core of a mucin molecule preferentially expressed by malignant cells. *Cancer Res* **51**: 2908–2916

Karanikas V, Hwang L, Pearson J, Ong C, Apostolopoulos V, Vaughan H, Xing P, Jamieson G, Pietersz G, Tait B, Broadbent R, Thynne G and McKenzie IFC (1997) Antibody and T cell responses of patients with adenocarcinoma immunized with mannan-MUC1 fusion protein. *J Clin Invest* **100**: 2783–2792

© Cancer Research Campaign 1999 

Karsten U, Diotel C, Klich G, Paulsen H, Goletz S, Mnller S and Hanisch FG (1998) Enhanced binding of antibodies to the DTR motif of MUC1 tandem repeat peptide is mediated by sit-specific glycosylation. *Cancer Res* **58**: 2541–2549

Lan ML, Hollingsworth MA and Metzger RA (1990) Polypeptide core of a human pancreatic tumor antigen. *Cancer Res* **50**: 2997–3001

McKenzie IFC and Xing P-X (1990) Mucins in breast cancer: Recent immunological advances. *Cancer Cells* **2**: 75–78

Nieves D, Henderson RA and Finn O (1995) Identification of an HLA-A2-restricted epitope from the tandem repeat domain of the epithelial tumor antigen mucin. *J Immunol* **155**: 4766–4774

Price MR (1988) High molecular weight epithelial mucin as markers in breast cancer. *Eur J Clin Oncol* **24**: 1799–1804

Price MR and Tendler SJB (1993) Polymorphic epithelial mucin (PEM): molecular characteristics and association with breast cancer. *The Breast* **2**: 3–11

Price MR, Hudecz F, O'Sullivan C, Baldwin RW, Edwards PM and Tendler SJB (1990) Immunological and structural features of the protein core of human polymorphic epithelial mucin. *Mol Immunol* **27**: 795–802

Sekine H, Ohno T and Kufe DW (1985) Purification and characterization of a high molecular weight glycoprotein detectable in human milk and breast carcinoma. *J Immunol* **135**: 3610–3615

Sell S (1990) Cancer associated carbohydrates identified by MoAbs. *Progress Pathol* **21**: 1003–1019

Soule HD, Vazquez J, Long A, Albert S and Brennan M (1973) A human cell line from a pleural effusin derived from a breast carcinoma. *J Natl Cancer Inst* **51**: 1409–1416

Stadie TRE, Chai W, Lawson AM, Byfield PGH and Hanisch FG (1995) Studies on the order and site specificity of GalNAc transfer to MUC1 tandem repeats by UDP-GalNAc: polypeptide *N*-acetylgalactosaminyltransferase from milk or mammary carcinoma cells. *Eur J Biochem* **229**: 140–147

Strauss GJ and Dekker J (1992) Mucin-type glycoproteins. *Crit Rev Biochem Mol Biol* **27**: 57–92

Taylor-Papadimitrou J, Petersen JA, Anklie J, Burchell J, Ceriani RL and Bodmer WF (1981) Monoclonal antibodies to epithelium specific components of the milk fat globule membrane: production and reaction with cells in culture. *Int J Cancer* **28**: 17–21

von Mensdorff-Pouilly S, Gourevitch MM, Kenemans P, Verstraeten AA, Litvinov SV, Van Kamp GJ, Meijer S, Vermorken J and Hilgers J (1996) *Eur J Cancer* **32A**: 1325–1331

Woolson RF (1987) *Statistical Methods for the Analysis of Biomedical Data*, pp. 17–24. John Wiley & Sons: New York

Xing PX, Prenzoska J, Quelch K and McKenzie IFC (1992) Second generation anti-muc 1 peptide MoAbs. *Cancer Res* **52**: 2310–2317

Zhang S, Graeber L, Helling F, Ragupathi G, Adluri S, Lloyd KO and Livingston PO (1996) Augmenting the immunogenocity of synthetic Muc1 peptide vaccines in mice. *Cancer Res* **56**: 3315–3319

 *© Cancer Research Campaign 1999*
